# Making Sense of Negative Findings from Mobile Attention Bias Modification Interventions for Individuals with Addictive Disorders: Quantitative Feasibility Study

**DOI:** 10.2196/16325

**Published:** 2019-11-12

**Authors:** Melvyn Zhang, Jiangbo Ying, Syidda B Amron, Zaakira Mahreen, Guo Song, Daniel SS Fung, Helen E Smith

**Affiliations:** 1 National Addictions Management Service Institute of Mental Health Singapore Singapore; 2 Department of Developmental Psychiatry Institute of Mental Health Singapore Singapore; 3 Family Medicine and Primary Care Lee Kong Chian School of Medicine Nanyang Technological University Singapore Singapore Singapore

**Keywords:** attention bias, cognitive bias, psychiatry

## Abstract

**Background:**

Advances in experimental psychology have led to a better understanding of unconscious, automatic processes that result in individuals relapsing into their substance-using habits. While some reviews have demonstrated the effectiveness of bias retraining of these unconscious biases, there have been other reviews that have highlighted that bias retraining is not always effective. Other studies have revealed there was no baseline biases among some participants. An examination of mobile bias retraining interventions has also revealed mixed results, with some reporting effectiveness and others null findings. A recent feasibility and acceptability study, done by the authors, revealed that 53% of participants have had no baseline biases and 21% of those with positive baseline biases did not have a positive change in magnitude following intervention.

**Objective:**

The aim of this paper was to explore potential variables (demographic and clinical) that could account for the negative baseline biases in the prior feasibility and acceptability study, and to discuss some of the factors that could account for the absence of baseline biases. We also explored potential reasons for why there was no reduction in the magnitude of attentional biases among individuals with baseline biases.

**Methods:**

Participants who were in the rehabilitation phase of their treatment were invited to participate. During the study they had to complete a set of baseline questionnaires, and on each day that they were on the ward they had to complete an attention bias assessment and modification task and rate their cravings using a visual analogue scale. Attention bias was deemed to be present if individuals had a positive score.

**Results:**

In our study, 53% (16/30) of individuals did not present with baseline attentional biases, and among those with positive baseline biases a total of 21% (3/14) of participants did not have a reduction in the overall magnitude of attentional biases. Chi-square analyses undertaken to compare the demographic characteristics of participants with and without baseline biases did not reveal any significant findings. However, with respect to clinical characteristics, those who had positive baseline biases had experimented with more substances.

**Conclusions:**

Our study is one of the first to have explored negative findings in attention bias modification interventions for individuals with addictive disorders. We postulate that several factors could account for the absence of baseline biases and there being no changes following bias retraining. Future research ought to take into consideration these factors.

## Introduction

Advances in experimental psychology over the years have led to a better understanding of unconscious, automatic processes [1] that result in individuals relapsing into their substance-using habits. Such processes include attentional biases and approach biases. Attentional biases refer to the automatic tendencies for attention to be preferentially allocated towards substance-related stimuli [2], whereas approach biases refer to the automated tendencies that result in individuals reaching out for substance stimuli [3]. The dual-process theoretical model explains that automatic biases develop as the chronic use of a substance results in increased automatic processing of the substance cue, with a resultant inhibition in the normal conscious cognitive control processes [4]. Tasks, such as the visual probe task and the Stroop task, are used in the assessment and modification of these automatic biases. The effectiveness of bias modification has been extensively evaluated. In a previous review, which considered studies involving participants with alcohol and tobacco use problems, bias modification interventions were deemed to be effective with a moderate effect size [5]. In a recent review from Heitman et al [6], they reported that only 10 of the identified 18 studies provided evidence for symptom change and effectiveness of attention bias modification. Of these 18 studies, 9 were focused on alcohol use, 6 were on nicotine use, and 3 were on opiate use [6]. Overall, the review found that multi-session interventions were more effective, especially for those who had adopted the alcohol attention control program [5].

Conventionally, attention bias modification interventions have been delivered in the laboratory, but advances in electronic and mobile health technologies have transformed the delivery of these interventions [7]. Zhang et al [8], in a review of a mobile cognitive bias intervention, reported that there had been at least 7 studies reporting that using the intervention with new technologies was more effective. However, one study (Robinson et al [9]) examining attention bias modification among smokers reported bias training to be ineffective, as the mobile intervention did not produce any changes in attentional biases. Similarly, Zhu et al [10], in their study examining the effectiveness of a mobile-based, computerized, cognitive addiction–therapy intervention, reported that their intervention led to no reduction in overall attention biases despite there being improvements in cognitive impairment and impulse control. In our recently published feasibility and acceptability study of a mobile attention bias intervention for inpatients with various addictive disorders (alcohol, opioids, cannabis, or stimulants) [11], we found that 16/30 participants (53%) did not have baseline attentional biases. Among those with baseline biases, attentional retraining did reduce the magnitude of the biases in all but three of the participants [11].

Negative findings are common in attention bias modification interventions. Negative findings are found in two contexts: at baseline assessment where there is an absence of biases, and when bias retraining fails to reduce the magnitude of attentional biases. Negative findings (the absence of effectiveness of bias modification) were highlighted in Heitman et al’s [6] review of the effectiveness of bias retraining for individuals with opioid use disorders. Sharpe [12], in his commentary, highlighted a recent study involving attention bias modification for pain in children which also had negative findings. In this study [13], participants did not present with baseline biases. One of the factors that Sharpe [12] highlighted was that the experimental procedure was not appropriate for children, and prior meta-analysis has highlighted the need to present stimulus of longer presentation rates [14] for these biases to be detected. Factors like the quantity and frequency of substance use and cravings could also affect the magnitude of attentional biases [15].

In our prior feasibility and acceptability study, there were at least 53% (16/30) of individuals who did not present with baseline biases, and of the participants with baseline biases, 21% (3/14) did not have a reduction of biases following bias intervention [10]. Thus, the aim of this paper is to explore potential variables (demographic and clinical) that could account for negative baseline biases, and to discuss some of the factors that could account for the absence of baseline biases. We also explored potential reasons for there to be no reduction in the magnitude of attentional biases among individuals with baseline biases. An exploration of these negative findings is pertinent, as it has a consequential implication on future attention bias research.

## Methods

### Overview

The methods for this study have been previously published as a study protocol [16] and in Zhang’s published feasibility and acceptability study [11]. Participants, who were in the rehabilitation phase of their treatment program at the National Addictions Management Service, Institute of Mental Health, were invited to participate. Participants who agreed to participate completed an informed consent form. The minimum recruitment target for the study was set at 30 participants.

### Ethical Approval

This study has been approved by the National Healthcare Group’s Domain Specific Research Board with the following reference number (2018/00316) on May 2, 2018.

### Inclusion and Exclusion Criteria

Patients were included in the study if they were aged between 21-65 years old, were diagnosed with a primary psychiatric disorder of alcohol, opioid, cannabis, stimulants, or polysubstance dependence, were diagnosed with polysubstance dependence, with alcohol, opioid, cannabis or stimulants as the main substance of use, were able to read and write in English, and were capable of using a smartphone or tablet device. Patients were excluded from the study if they had a known history of cognitive impairment or dementia, if they had a history of seizures or a prior history of withdrawal seizures, if they had a history of migraines triggered by flashing lights, and if they had any moderate to severe comorbid psychiatric disorders based on clinical assessment.

### Measures

Upon recruitment into the study, baseline demographic and clinical information were acquired from the participants, including: nationality, gender, marital status, race, religion, highest level of education, housing conditions, current substance use, previous treatment history, chronic diseases (psychiatric or physical disorders), and any current psychiatric medication use. Participants also completed the modified Addiction Severity Index (ASI)–Lite, the Severity of Drug Dependence Scale (SDS), and the Short Form (SF-12) questionnaires. The ASI-Lite collects information about the participants’ drug and alcohol use. All participants were asked about their alcohol and substance use in the last 30 days, in the last last month, and over their lifetime. The SDS questionnaire has 5 questions, all of which are focused on the psychological components of dependence. The SF-12 assessed the participants’ self-reported quality of life.

Upon the completion of these questionnaires, participants were oriented on how they should use the application. On the first day of the intervention, participants were required to undertake an assessment task, then an intervention task, and then rest for 15 minutes before completing another assessment task. On the subsequent days of the intervention, participants were asked to complete an intervention task and then rest for 10 minutes before completing the assessment task. All participants were also asked to complete a visual-analogue craving scale before and upon the completion of each of the bias modification tasks. Participants were to undertake the tasks only if it was a weekday and were exempted from undertaking these tasks on weekends or public holidays.

In the assessment task, participants were presented with a central fixation cross for 500 milliseconds. Following the disappearance of the fixation cross, they were presented with a set of images, with one image being a substance image and the other a neutral image. The images would then disappear, and a probe would replace the position of one of these images. Participants were instructed to indicate the position of the probe by pressing on buttons within the application as rapidly as they could. In the assessment task, the probe would replace the substance-related image and the neutral image equally. In the intervention task, the probe would always replace the neutral image for the successful retraining of the attentional processes.

### Statistical Analyses

Data collated was analyzed using SPSS Version 22 (IBM Corp, Armonk, New York, United States). Baseline demographic information of the subjects was summarized using descriptive statistics. The presence of attentional biases was determined based on the mean reaction times taken to respond to the position of the probes that replaced either the drug or neutral stimuli. The formula used for the computation of attentional biases was 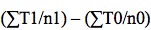, where T1 refers to the time for probes that replaced the neutral stimulus, n1 refers to the number of trials for probes that replaced the neutral stimulus, T0 refers to the time for probes that replaced the substance stimulus, and N0 refers to the number of trials for probes that replaced the substance stimulus. A chi-square test was conducted to compare the demographic characteristics of those with and without baseline attentional biases.

## Results

### Summary

40 participants were screened and invited to participate in the study, of which 10 declined, thus leaving 30 who participated. A total of 11 of these participants failed to complete all the planned interventions, 10 of whom elected for premature discharge from the ward and 1 who withdrew from the study following the initial intervention. Table 1 (previously published in Zhang et al’s feasibility and acceptability study) [11] provides an overview of the baseline attention bias scores for each of the participants and their change in scores across time.

### Baseline Attentional Biases

It is evident from Table 1 that only 14/30 participants presented with positive attentional biases at baseline. The remaining 16 participants did not have any underlying attentional biases.

### Changes in Attentional Biases Over Time for Individuals With Positive Baseline Biases

Among those with baseline attention biases, there was a general decrease in their attentional bias scores from baseline till the end of the intervention, except for three participants (numbers 8, 19, and 20). The changes in attentional biases scores ranged from 12.0 to 409.5 milliseconds.

Participants with and without baseline attentional biases were compared. Table 2 provides an overview of the baseline demographic characteristics of participants with and without attentional biases at baseline. The mean age of those with positive baseline attention bias was 46.3 years old, while those without attention bias had a mean age of 45.8 years. In terms of substance use, among those with positive baseline attention bias, 3 were diagnosed with alcohol use, 9 with opioid use, and 2 with stimulant use. Among those without baseline attention bias, 3 were diagnosed with alcohol use, 8 with opioids use, 2 with cannabis use, and 3 with stimulant use. Most of the participants were Singaporean. There was one female in the positive group and three females in the negative group. Most of the participants had a primary or secondary school education, and most were unemployed. Physical disorders were more common (43.8%) among those with negative baseline attention bias. The mean scores for the severity of substance dependence were comparable (10.93 for the positive group and 10.31 for the negative group).

**Table 1:** Overview of baseline attention bias scores and changes in scores over time.

Participant	Drug	Baseline	Post first session	Post second session	Post third session	Post fourth session	Post fifth session	Overall change in attentional bias
001	Stimulants	30.3	70.6	36.3	9.3	–23.6	13.3	17.0
002^a^	Stimulants	–22.4	–23.4	–11.7	—^b^	—	—	10.7 (increased)
003	Stimulants	6.7	–3.6	–28.9	–11.3	4.1	–7.3	14.0
004^c^	Opioid	32.1	28.7	12.2	31.2	20.1	—	12.0
005^c^	Alcohol	91.2	–23.3	–37.4	–5.3	–33.2	—	124.4
006^c^	Opioid	98.9	33.4	14.5	–9.7	–31.5	—	130.4
007	Stimulants	–30.5	–13.5	–23.4	–27.7	–28.2	–7.6	22.9 (increased)
008^a^	Opioids	58.7	85.1	—	—	—	—	26.4 (increased)
009^a^	Opioids	25.8	13.5	—	—	—	—	12.3
010	Cannabis	–9.9	–20.7	–54.6	–14.1	–22.9	–48.4	38.5
011	Opioids	–30.9	2.4	–15.4	–7.4	14.2	7.3	38.2 (increased)
012^a^	Opioids	0.7	34.8	–15.3	–52.4	—	—	53.1
013	Alcohol^d^	–20.9	–12.3	—	—	–75.8	–48.6	27.7
014^a^	Alcohol	397.7	44.2	45.3	–32.0	–11.7		409.4
015	Cannabis	–7.7	–40.5	8.6	–33.6	–31.8	–50.3	42.6
016^a^	Opioids	–27.4	—	—	—	—	—	—
017	Opioids	–42.5	–64.8	63.4	8.9	–15.5	26.7	69.2 (increased)
018	Opioids	27.9	–17.8	3.2	–22.6	–104.6	–9.2	37.1
019^a^	Opioids	3.8	35.1	13.7	32.4			28.6 (increased)
020	Opioids	10.1	9.4	105.2	54.1	–1.7	20.3	10.2 (increased)
021	Alcohol	224.5	61.5	73.3	176.7	130.3	107.0	117.4
022	Opioids	–52.9	10.9	5.6	39.1	36.5	76.6	129.6 (increased)
023	Opioids	–36.4	18.0	45.0	74.8	35.9	41.3	77.7 (increased)
024^a^	Opioids	–16.9	45.2	1.49	3.8	33.9	—	50.8 (increased)
026^a^	Opioids	–77.1	–82.5	—	—	—	—	5.4
027	Stimulants	–15.2	–11.6	10.1	–27.0	–25.7	–1.6	13.6 (increased)
028	Alcohol	–33.3	–29.4	–48.3	–10.9	–15.6	–11.6	21.8 (increased)
029^a^	Alcohol	–41.4	–18.0	20.6	28.2	—	—	69.6 (increased)
030	Opioids	1.3	38.6	7.9	11.5	13.0	–8.9	10.2
031^a^	Opioids	–38.4	–282.7	–166.4	–190.1	—	—	151.6

^a^Participants who did not complete the study as they left the voluntary program.

^b^Not applicable.

^c^There was a holiday during the assessment period and thus the maximum number of sessions completed was 4.

^d^Due to a technical issue, Participant 13 was not administered an assessment task following the second intervention so the participant took another intervention task instead. Attentional bias assessment was performed only after the fourth session.

**Table 2:** Baseline demographic characteristics (n=30).

Demographic characteristics		Positive baseline attention bias (n=14)	Negative baseline attention bias (n=16)
Age (years), mean (SD)		46.3 (12.7)	45.8 (10.7)
**Substance use, n (%)**			
	Alcohol	3 (21)	3 (19)
	Opioids	9 (64)	8 (50)
	Cannabis	0 (0)	2 (13)
	Stimulants	2 (14)	3 (19)
**Nationality, n (%)**			
	Singaporean	12 (86)	15 (94)
	Others	2 (14)	1 (6)
**Gender, n (%)**			
	Male	13 (93)	13 (81)
	Female	1 (7)	3 (19)
**Race, n (%)**			
	Chinese	4 (29)	3 (19)
	Malay	6 (43)	7 (38)
	Indian	3 (21)	6 (38)
	Others	1 (7)	1 (6)
**Education, n (%)**			
	Primary education	4 (29)	3 (19)
	Secondary education	7 (50)	9 (56)
	Junior college or polytechnic/technical studies	1 (8)	4 (25)
	Undergraduate studies	2 (14)	0 (0)
**Employment, n (%)**			
	Unemployed	10 (71)	13 (81)
	Part-time employment	1 (8)	2 (13)
	Full-time employment	3 (21)	1 (6)
**Housing, n (%)**			
	Homeless	4 (29)	2 (13)
	1 room	4 (29)	4 (25)
	2 rooms	2 (14)	0 (0)
	3 rooms	1 (7)	2 (13)
	4 rooms	0 (0)	7 (44)
	5 rooms	2 (14)	0 (0)
	Others	1 (7)	1 (6)
Psychiatric disorders, n (%)		2 (14)	1 (6)
Physical disorders, n (%)		4 (29)	7 (44)
Severity of substance dependence scores, mean (SD)		10.93 (3.33)	10.31 (3.28)

Using chi-square (χ^2^) analysis to compare the categorical demographical variables amongst those with and without baseline attentional biases, we found no significant differences between the two groups with respect to nationality (χ^2^_1_=0.536; *P*=.46), gender (χ^2^_1_=0.871; *P*=.35), substances (χ^2^_3_=1.875; *P*=.60), race (χ^2^_3_=1.014; *P*=.80), education status (χ^2^_3_=4.078; *P*=.25), employment (χ^2^_2_=1.598; *P*=.45), accommodation/housing (χ^2^_6_=11.92; *P*=.06), psychiatric diagnoses (χ^2^_1_=0.536; *P*=.46) and physical diagnosis (χ^2^_1_=1.916; *P*=.38).

Table 3 and Table 4 provide an overview of participants with and without baseline attentional biases and their reported drug use, recorded on the modified ASI-Lite questionnaire. As evident from Table 2, Table 3, and Table 4, both the two participants with cannabis use disorder did not present with any baseline attentional biases. Most of the participants had started using their substance of abuse at a young age, and most of them were using daily prior to admission. Individuals in the group with positive baseline attention bias had experimented with more substances, as compared to individuals in the group without baseline attention bias.

**Table 3:** Substance use history of participants with positive baseline attention bias.

Participant	Age	Baseline bias	Current drug	Drug history	Age of onset	Age of problematic use	Frequency prior to admission
001	46	30.3	Stimulants	Polysubstance (9)^a^	45	45	Daily (1-2 grams, once per day)
003	35	6.7	Stimulants	Polysubstance (7)	31	31	Daily (0.5 grams, once per day)
004	46	32.1	Opioids	Polysubstance (12)	14	17	Daily (30-40 mL, 1-2 times per day)
005	26	91.2	Alcohol	Alcohol (1)	20	21	Daily (750 mL, 2-3 times per day)
006	64	98.9	Opioids	Polysubstance (11)	20	22	Daily (2 Straws, once daily)
008	60	58.7	Opioids	Polysubstance (4)	13	13	Daily (1 Straw, 2 times daily)
009	28	25.8	Opioids	Polysubstance (5)	17	19	Daily (1.5 straws, 6 times daily)
012	28	0.7	Opioids	Polysubstance (5)	18	25	Daily (5 tablets, 5 times daily)
014	61	397.7	Alcohol	Polysubstance (5)	13	27	Daily (3 tall cans, 2 times daily)
018	54	27.9	Opioids	Polysubstance (5)	34	34	*Patient did not fill up
019	57	3.8	Opioids	Polysubstance (3)	40	40	Daily (90 mL, once per day)
020	45	10.1	Opioids	Polysubstance (4)	23	23	Daily (1 straw, 3 times daily)
021	50	224.5	Alcohol	Alcohol & tobacco (2)	15	50	Daily (8 cans, once daily)
030	48	1.3	Opioids	Polysubstance (3)	16	16	Daily (4 Straws, 4 times daily)

^a^(n): This refers to the total number of substances the individual has experimented with.

**Table 4:** Substance use history of participants with negative baseline attention bias

Participant	Age	Baseline bias	Current drug	Drug history	Age of onset	Age of problematic use	Frequency prior to admission
002	44	–22.4	Stimulants	Polysubstance (4)^a^	38	40	Daily (Unknown quantity, 6 times daily)
007	32	–30.5	Stimulants	Polysubstance (3)	29	30	Daily (0.3 grams, 5 times daily)
010	59	–9.9	Cannabis	Polysubstance (5)	59	59	Daily (2 joints once per day)
011	50	–30.9	Opioids	Polysubstance (3)	24	24	Daily (4 straws, 4 times per day)
013	42	–20.9	Alcohol	Alcohol and tobacco (2)	18	18	Daily (3-4 bottles, 3 times per day)
015	57	–7.7	Cannabis	Polysubstance (7)	57	57	Daily (1 joint, 20 times per day)
016	47	–27.4	Opioids	Polysubstance (6)	17	17	Daily (1 joint, 6-7 times per day)
017	63	–42.5	Opioids	Polysubstance (4)	16	17	Daily (0.5 straws, 3 times per day)
022	43	–52.9	Opioids	Polysubstance (6)	16	17	Daily (2 straws, 2 times per day)
023	54	–36.4	Opioids	Polysubstance (6)	15	16	Daily (3 straws, 10 times per day)
024	46	–16.9	Opioids	Tobacco & heroin (2)	17	21	Daily (7 straws, 4 times per day)
026	57	–77.1	Opioids	Tobacco & heroin (2)	17	28	Daily (1 straw, 2 times per day)
027	31	–15.2	Stimulants	Polysubstance (9)	17	25	Daily (0.3 grams throughout the day)
028	44	–33.3	Alcohol	Alcohol and tobacco (2)	17	28	Daily (750 mL, 16-18 times per day)
029	39	–41.4	Alcohol	Polysubstance (3)	14	30	Daily (9-10 cans, 9 times per day)
031	25	–38.4	Opioids	Polysubstance (8)	20	20	Daily (1 straw, 2 times per day)

^a^(n): This refers to the total number of substances the individual has experimented with.

## Discussion

### Primary Findings

We are unaware of any other study that has examined negative findings in attention bias modification intervention for individuals with addictive disorders. In our study, 53% (16/30) of individuals did not present with baseline attentional biases, and among those with positive baseline biases, 21% (3/14) of participants did not have a reduction in the overall magnitude of attentional biases. Chi-square analyses undertaken to compare the demographic characteristics of participants with and without baseline biases did not reveal any significant findings. However, with respect to clinical characteristics, those who had positive baseline biases had experimented with more substances. We postulate that there are four factors that could account for the absence of baseline biases: the period of abstinence, the images used in the mobile application, the stimulus timings, and the clinical characteristics of the participants. With regards to the lack of retraining effects, we theorize that this might be due to practice effects or to the novelty of the other neutral stimulus.

The lack of baseline biases we observed may be due firstly to the fact that our participants had received treatment and had been abstinent for at least seven days prior to being recruited into the study. This period of abstinence might have affected their baseline attentional biases. In most of the published studies [17,18], participants who had been recruited were in the detoxification phase of treatment. In our study, participants were considered for the intervention only upon the successful completion of their detoxification program. Participants who were in their detoxification phase were not recruited, as we anticipated that them having withdrawal symptoms might affect their ability to undertake the required interventions. The existing medication-assisted detoxification was highly effective in ameliorating attentional biases among these participants. Psychotropic medications prescribed in the acute detoxification phase might have affected or reduced attentional biases. Dopaminergic and serotonergic agents (such as antipsychotics and antidepressants) could have reduced attention biases in the acute phase, as highlighted in Zhang et al’s [19] prior review. Some of these medications might have been used for symptomatic relief of psychotic or affective symptoms in these individuals during the withdrawal phase. Unfortunately, in this current study we did not request ethical approval to extract information from the medical records, and thus the association between the prescription of medications and attentional biases cannot be determined.

Secondly, the nature of the images/stimuli used in our application may have contributed to a null finding at baseline assessment. The images used in the application may not be good representations of the substances participants were familiar with, and thus did not manage to capture their attention. This is in line with Field et al’s [20] report that one of the key factors leading to the poor reliability of the visual probe task is that of the nature of the stimulus used. Field et al [20] highlighted the importance of personalization of the stimulus presented to the participants, as it is postulated that a stimulus that is relevant and identifiable to the participant could increase their baseline attentional bias score and provide evidence of greater change in the magnitude of attentional biases. Many of the images included in the mobile application were extracted from the internet via the United States Drug Enforcement Agency media library. Fewer images came from Singapore’s Central Narcotics Bureau’s website. It might be possible that the images included do not approximate the real object and are not realistic enough for participants.

Thirdly, as highlighted by Sharpe [12], the experimental procedure that we used in our study might have affected the detection of baseline biases, in regard to the duration of the presentation of the images. In our study, we presented the stimulus for 500 milliseconds, like most prior studies which had evaluated the reliability of the visual probe task [21,22]. We are aware that there have been other studies, such as those by Constantinou et al, Frankland et al, and two by Garland et al [21-24], that have presented the stimulus for as little as 200 milliseconds to as long as 1500 to 2000 milliseconds. It has been previously postulated in the literature that the short stimulus interval helped in the evaluation of automatic orientating tendencies, whereas the long stimulus interval helped in the evaluation of controlled attentional processing. In some of these previous studies [23-26], the stimulus was being presented to individuals at both a short and long stimulus interval. The fact that we had a fixed stimulus timing interval of 500 milliseconds might have resulted in us not capturing potentially controlled attentional processes, which might account for individuals not having baseline biases. It is thus of importance for there to be a review of stimulus timings that have been previously used and to correlate the timings with the effectiveness of bias detection and modification.

Lastly, with regards to the absence of baseline biases, Dean et al’s [15] research has highlighted that clinical characteristics of participants modulate their baseline attentional biases. In our study, we did not manage to find any demographic variables that could account for the differences among those with baseline biases and those without. There were, however, more individuals in the group with positive baseline biases that had previously abused a larger quantity of substances. This observation is congruent with previous research, which reported that the frequency and quantity of drug use do moderate the magnitude of the attentional biases. Field et al [21], in their previous study, reported that there were more robust attentional biases among individuals who were heavy drinkers when the alcohol images were presented for 500 and 2000 milliseconds, respectively. In another study by Noel et al [27], the authors reported that there was an absence of attentional biases among those who were abstinent from alcohol use. It is evident from these studies that the amount of substance use affects the attentional biases, and this should be an important consideration when planning for future attention bias modification interventions for individuals with addictive disorders since there may be individuals who are abstinent and without baseline biases, so attention bias modification might not be appropriate for them. It is important that future studies consider evaluating individuals for baseline biases before administration of bias modification.

Regarding the negative findings among those with positive baseline attentional biases (ie, no impact of attentional retraining), we postulate that the absence of effects could be accounted for by practice effects. Thus, for some individuals, over the course of the intervention they have learnt to focus only on the position of the probe and not on the stimulus. There is also the possibility that the neutral stimulus might have appeared to be more novel and appealing to some individuals, which lead to an increased focus on the neutral stimulus and a resultant more rapid response in identifying probes that replace the neutral stimulus.

### Conclusions

Our study is one of the first to have explored negative findings in attention bias modification interventions for individuals with addictive disorders. We postulate that there could be several factors that could account for the absence of baseline biases and there being no changes following bias retraining. Future research ought to take into consideration these factors, and it is especially pertinent for clinical studies to evaluate for baseline bias prior to administration of bias retraining interventions.

## Abbreviations

ASI: Addiction Severity Index
SDS: Severity of Drug Dependence Scale
SF-12: Short Form 12-item questionnaire
